# Energy and Entropy in Turbulence Decompositions

**DOI:** 10.3390/e21020124

**Published:** 2019-01-29

**Authors:** Václav Uruba

**Affiliations:** 1 Department of Fluid Dynamics, Institute of Thermomechanics of the Czech Academy of Sciences (CAS), v. v. i., Dolejškova 5, 18200 Praha 8, Czech Republic; uruba@it.cas.cz; Tel.: +420-266-053-414; 2 Faculty of Mechanical Engineering, Department of Power System Engineering, University of West Bohemia, Universitní 22, 30614 Plzeň, Czech Republic

**Keywords:** turbulence, coherent structures, decomposition, energy, entropy

## Abstract

The role of energy and entropy in the decomposition of turbulent velocity flow-fields is shown in this paper. Decomposition methods based on the energy concept are taken into account—proper orthogonal decomposition (POD) and its extension bi-orthogonal decomposition (BOD). The methods are well known; however, various versions are used and the interpretation of results is not straightforward. To make this clearer, the specific definition of modes is suggested and specified; moreover, energy- and entropy-motivated views on the decomposed modes are presented. This concept could offer new possibilities in the physical interpretation of modes and in reduced-order modeling (ROM) strategy efficiency evaluation.

## 1. Introduction

Turbulence in fluids is a highly dynamical phenomenon which is characterized by complex topology. The duality of randomness and coherency is typical and can be identified as a blend of random behavior of coherent structures. It is extremely important to distinguish between these two complementary parts. In general, in this extremely difficult task, the quantities of “coherency” and “entropy” are of great importance.

The turbulent flow-field is populated by coherent structures (e.g., see Reference [1]). These are vortices very often of various sizes, positions, and orientations in space with complex dynamics. To study this complicated phenomenon, several strategies are used. The classical approach is to identify the individual structures and explore their evolution in time and space. This is a very exhausting task; thus, statistical tools are applied. The basic method of coherent structure identification used is proper orthogonal decomposition (POD) [2] or its generalized version bi-orthogonal decomposition (BOD) [3].

The POD and BOD methods are currently used very often for representation dynamics of nearly any quantity field. However, the definition and the implementation of the methods are not unique. Some approaches consider the fluctuation part of Reynolds decomposition only, while others use the instantaneous values including the mean value. These different strategies lead to different results, and, of course, the meaning of the resulting modes is also different.

The physical interpretation of the modes remains a crucial unresolved point of the decomposition methods. The presented paper shows specific definitions of the methods and links the results to quantities of turbulent kinetic energy and information entropy. This insight helps in understanding the physical meaning of the results.

The turbulent data are often available in the form of snapshots. A snapshot shows the instantaneous spatial distribution of a given physical quantity characterizing the flow-field. Instantaneous velocity vector-fields are usually used. However, the methods could work with any physical quantity whose distribution in space can be determined, e.g., vorticity, pressure, or concentration of a contaminant; both scalar and vector fields are allowed. The input data can be obtained from experiments, e.g., from particle image velocimetry (PIV) or other optical methods or from mathematical modeling, direct numerical simulation, or large eddy simulation. In this paper, we focus on velocity vector fields.

The decomposition methods are based on the idea of Hilbert space, which is defined by the set of all snapshots forming a natural basis of the Hilbert space (e.g., see Reference [4]). The goal of the decomposition methods is to find another appropriate base with a distinct physical meaning. The POD and BOD methods look for an orthonormal basis maximizing the dynamic data variance. Variance could be linked to fluctuation energy. The orthogonality condition implies decorrelation of the modes, which is necessary but not sufficient for statistical independence (e.g., see Reference [5]).

The decomposition methods based on POD could provide an efficient basis for reduced-order modeling (ROM) of complex system dynamics. The searching process for the optimal basis covering the maximal fluctuating kinetic energy of real system dynamics can be considered as an information compression process, if the information describes the spatio-temporal behavior of the system. However, not all information can be compressed efficiently; thus, the efficiency of this process needs to be assessed. The more coherent the system behavior is, the more efficient the compression will be. The efficiency of the compression can be assessed with help of system information entropy evaluation. As the level of entropy increases, the compression process becomes less efficient. Entropy is distributed in time and space as energy is, while the global information is provided by total or global entropy.

In the presented paper, the POD and BOD methods are introduced in the context of coherency evaluation, supplemented with the definition of entropy.

## 2. Historical Notes

The POD method has applications in almost any scientific field where extended dynamical systems are involved. This fact accounts for the frequency of POD’s discovery. We could follow this process on the time axis: first, Pearson in 1901 [6], then Hotelling in 1933 [7], Kosambi in 1943 [8], Loeve in 1945 [9], Karhunen in 1946 [10], Pougachev in 1953 [11], and Obukhov in 1954 [12]. All of these scholars were credited with the independent discovery of POD under one of its many titles, which include principle component analysis, Karhunen–Loève decomposition or expansion, principle factor analysis, Hotelling transform, and collective coordinates. Details of these processes can be found in Reference [13].

Recently, POD was widely used in studies of turbulence, as appropriate data are at our disposal. Historically, the POD method was introduced in the context of turbulence by Lumley in 1967 [2] as an objective definition of what was previously called big eddies, now widely known as coherent structures.

POD is considered to be a natural idea to replace the usual Fourier decomposition in inhomogeneous directions. Adrian et al. [14] considered POD as inhomogeneous filtering applied on flow data in the framework of the large eddy simulation method. Classical homogeneous filtering, using the Gaussian filter for example, is inconsistent with the fact that the turbulent eddies increase in size as they move away from the wall. This problem can be addressed using the POD method to construct low-pass filters that are inhomogeneous in one or more directions. POD provides an optimal set of base functions for an ensemble of data in the sense that it is the most efficient way of extracting the most energetic components of an infinite dimensional process with only a few modes.

The POD method is optimal in the sense that the series of eigenmodes converges more rapidly (in quadratic mean) than any other representation. Convergence is very fast in flows in which large coherent structures contain a major fraction of the total kinetic energy. As an example, the pseudo-periodical vortex streets in wakes or strong shear layers can be mentioned—see the example in Section 6.

An advantage of this method is its objectivity and lack of bias. Given a realization of an inhomogeneous, energy-integrable velocity field, it consists of projecting the random field on a candidate structure, and selecting the structure which maximizes the projection in the quadratic mean. In other words, we are interested in the structure which is best correlated with the random, energy-integrable field. More precisely, we are given an ensemble of realizations of the field, and the purpose is to find the structure which is best correlated with all the elements of the ensemble. Thus, we want to maximize a statistical measure of the magnitude of the projection, which can be given by the mean square of its absolute value.

The calculus of variations reduces this problem of maximization to a Fredholm integral equation of the first kind, whose symmetric kernel is the autocorrelation matrix (e.g., see Reference [4]). The properties of this integral equation are given by Hilbert–Schmidt theory. There is a denumerable set of eigenfunctions (structures). The eigenfunctions form a complete orthogonal set, which means that the random field can be reconstructed. The coefficients are uncorrelated, and their mean-square values are the eigenvalues themselves. The kernel can be expanded in a uniformly and absolutely convergent series of the eigenfunctions and the turbulent kinetic energy is the sum of the eigenvalues. Thus, every structure makes an independent contribution to the kinetic energy and Reynolds stress.

## 3. Decomposition Methods

The existence of so-called coherent structures in turbulent flows is now well accepted (e.g., see Reference [1]). Lumley [15] introduced the concept of “building blocks” (i.e., the basis of non-specified functions) based on the notion of “energetic modes” on which the velocity field is projected. The modes maximize velocity variance, i.e., the kinetic energy of the process. 

Extraction of deterministic features from a random, fine-grained turbulent flow is a challenging problem. Lumley proposed an unbiased technique for identifying such structures. The method consists of extracting the candidate which is best correlated, in a statistical sense, with the background velocity field. The different structures are identified with the orthogonal eigenfunctions of the decomposition theorem of probability theory. The POD method is, thus, a systematic way of finding organized motions in a given set of realizations of a random field. However, the interpretation of individual POD modes is not straightforward.

In the last few decades, the POD method was used extensively to study flow-field dynamics. A few examples are described here. In environmental aerodynamics, POD is often used; an overview of POD applications in wind engineering can be found in Reference [16]. In Reference [17], POD applications in biomechanics are described, particularly in blood flow dynamics analysis. Another example from the field of vibro-acoustics can be found in Reference [18]. Many other examples can be found in literature.

There are numerous variants of the decomposition methods. Many of them are based on the POD concept; however, there are some methods which use a different principle.

One of the concepts that developed the POD philosophy is so-called “extended POD” (see Reference [19]); it combines velocity and any other physical quantity in one process resulting in a feature tracking method. Another modification is called “spectral POD” (see Reference [20]), combining analysis in real and spectral spaces to extract information on energy and frequencies. The POD method can be applied not only to the data analysis process, but also to mathematical modeling (e.g., see Reference [21]). POD can also be combined with some other concepts, e.g., with wavelet transformation (e.g., see References [22,23]).

POD is used as a basic support method in several reduced-order modeling strategies. POD defines the basis for the Galerkin method (e.g., see References [24,25]).

Not all decomposition methods are based on the energy concept; there are also methods based on stability assessment, such as dynamic mode decomposition (e.g., see Reference [26]) or oscillation pattern decomposition (e.g., see Reference [27]). These methods are appropriate to study the presence of individual frequencies and topologies connected in spatio-temporal data. 

An overview of energy-based decomposition methods can be found in Reference [28]. In the present paper, we are not concerned with those special methods.

### 3.1. Definition of Kinetic Energy 

Kinetic energy is a key quantity in flow-field analysis. Specific kinetic energy is often considered related to a unity mass. In the theory of turbulence, it is based on a definition of velocity subjected to Reynolds decomposition, as follows:

(1) $$v_{i}\left(x,t\right)=V_{i}\left(x\right)+v_{i}^{\prime }\left(x,t\right),$$

where $v_{i}$ is the instantaneous *i*-th velocity component, $V_{i}$ is the velocity component mean value with $v_{i}^{\prime }$ as its fluctuation part, x is a position vector, and t is time. The mean value is evaluated as an ensemble average $\langle v_{i}\left(x,t\right)\rangle$, usually replaced by the time mean value $\frac{1}{T}\int_{0}^{T}v_{i}\left(x,t\right)\;dt$, as the process is supposed to be ergodic. T is the time interval, which is sufficiently long.

Then, the total kinetic energy $K_{T}$ can be defined as the sum of mean kinetic energy K and fluctuating kinetic energy k, as shown in Equation (2).

(2) $$K_{T}\left(x\right)=\frac{1}{2}\langle v_{i}^{2}\left(x,t\right)\rangle =\frac{1}{2}\left(V_{i}^{2}\left(x\right)+\langle {v_{i}^{\prime }}^{2}\left(x,t\right)\rangle \right)=K\left(x\right)+k\left(x\right),\;i=1,2,3.$$

The fluctuating kinetic energy k is called turbulent kinetic energy (TKE).

(3) $$k\left(x\right)=\frac{1}{2}\langle {v_{i}^{\prime }}^{2}\left(x,t\right)\rangle ,\;i=1,2,3.$$

The index i is additive, subjected to the Einstein rule in Equations (2) and (3).

From an experiment, n velocity components (typically 2 or 3) in a rectangular region can be evaluated for overall M points in space. The *i*-th component of the *j*-th point in space is $v_{ij}\left(t\right)$, the corresponding position vector is $x_{j}$, and $v_{ij}^{\prime }\left(t\right)$ is the velocity fluctuation. The velocity fluctuating components $v_{ij}^{\prime }\left(t\right)$ can be reordered into a single-state vector of velocity fluctuations u(t) or $u_{i}\left(t\right)$ in tensor notation. Now, we define the kinetic energy of the system E as a sum of energies of all detected components in all points in space.

(4) $$E=\frac{1}{M}\sum_{j=1}^{M}k\left(x_{j}\right)=\frac{1}{2M}\sum_{i=1}^{n}\sum_{j=1}^{M}\langle {v^{\prime }}_{ij}^{2}\left(t\right)\rangle =\frac{1}{2M}\sum_{i=1}^{N_{x}}\langle u_{i}^{2}\left(t\right)\rangle ,\;N_{x}=n\cdot M.$$

The total number of velocity components $N_{x}$ involved in the problem is equal to the number of degrees of freedom, which is also the dimension of the state vector.

This total kinetic energy of the system is taken into account in subsequent analysis based on the energy approach. Please note that, for the two evaluated velocity components, only part of the TKE is covered by this quantity.

### 3.2. Proper Orthogonal Decomposition

Lumley proposed to define a coherent structure with functions of the spatial variables that have maximum energy content. That is, coherent structures are φ(x)’s (or linear combinations of) which maximize the following expression

(5) $$\frac{\langle {\left(\varphi \left(x\right),u\left(x,t\right)\right)}^{2}\rangle }{\langle \varphi \left(x\right),\varphi \left(x\right)\rangle },$$

where the expression (f,g) denotes the inner product $\int_{\Omega }fg\;d\Omega$ in $L^{2}$ on the space domain Ω, and 〈f〉 is the mean in time. Thus, if φ(x) maximizes Equation (5), it means that, if the flow-field is projected along φ(x), the average energy content is larger than if the flow-field is projected along any other structure. Then, in the space orthogonal to the evaluated φ(x), the maximization process can be repeated, and, in this way, a whole set of orthogonal functions $\varphi_{i}\left(x\right)$ can be determined. The power of POD lies in the fact that the decomposition of the flow-field on the basis of POD eigenfunctions converges optimally fast in the $L_{2}$-sense.

Using variation calculus, it can be shown that a necessary condition for φ(x) to maximize Equation (5) is that it is the solution of the following Fredholm integral equation of the second type:

(6) $$\int_{\Omega }R_{s}\left(x,x^{\prime }\right)\;\varphi \left(x^{\prime }\right)dx^{\prime }=\lambda^{2}\;\varphi \left(x\right),$$

where Ω is the flow domain and $R_{s}$ is the space-correlation matrix, as shown in Equation (7).

(7) $$R_{s}\left(x,x^{\prime }\right)=\langle u\left(x\right)\;u^{T}\left(x^{\prime }\right)\rangle =\int_{T}u\left(x,t\right)\;u^{T}\left(x^{\prime },t\right)\;dt.$$

The correlation matrix is symmetric and positive definite. According to Hilbert–Schmidt theory, Equation (6) has a denumerable set of orthogonal solutions, i.e., eigenfunctions $\varphi_{i}\left(x\right)$ with corresponding real and positive eigenvalues $\lambda_{i}$.

The eigenfunctions are orthogonal and can be normalized as follows:

(8) $$\left(\varphi_{i},\varphi_{j}\right)=\delta_{ij}.$$

The closure of the span of POD eigenfunctions is equal to the set of all realizable flow-fields. Therefore, any flow-field can be expressed as a linear combination of the eigenfunctions.

(9) $$u\left(x,t\right)=\sum_{k=1}^{\infty }a_{k}\left(t\right)\;\varphi_{k}\left(x\right).$$

Equation (9) represents the continuous variant of POD implementation. However, this equation is not very appropriate for direct application.

The eigenvalues $\lambda_{i}^{2}$ are related to the given mode energy, as they represent a sum of all velocity component variances.

### 3.3. Snapshot POD

This variant of the POD method’s implementation uses space instead of time correlations.

The method was proposed by Sirovich in 1987 [29]. For snapshot POD, we need a set of $N_{t}$ snapshots $u_{i}\left(x\right)$ of the fluctuating velocity field. The snapshots are taken at different times from a simulation.

(10) $$u_{k}\left(x\right)=u\left(x,t_{k}\right).$$

The snapshots should be mutually linearly independent; this condition implies that $N_{t}\leq N_{x}$. The maximization problem in Equation (5) can be reformulated for the snapshots as follows:

(11) $$\frac{\frac{1}{N_{t}}\sum_{k=1}^{N_{t}}{\left(\varphi \left(x\right),u_{k}\left(x\right)\right)}^{2}}{\left(u_{k}\left(x\right),u_{k}\left(x\right)\right)}.$$

Supposing applicability of the ergodicity hypothesis, we can rewrite the expression for the correlation function in the following way:

(12) $$R_{s}\left(x,x^{\prime }\right)=\underset{k\to \infty }{\lim}\sum_{k=1}^{N_{t}}u_{k}\left(x\right)\;u_{k}^{T}\left(x^{\prime }\right).$$

In this equation, the time between the snapshots has to be large enough for the snapshots to be uncorrelated. The idea is now to take a finite N large enough for a reasonable approximation of $R_{s}\left(x,x^{\prime }\right)$. Substituting Equation (12) into the Fredholm integral Equation (6) results in a degenerate integral equation. Therefore, the solutions are linear combinations of the snapshots as follows:

(13) $$\varphi_{k}\left(x\right)=\sum_{k=1}^{N_{t}}q_{ki}u_{k}\left(x\right).$$

Thus, the problem is reduced to finding the coefficients $q_{ki}$ of the linear combination. If we substitute Equation (13) into the degenerated integral equation, we obtain the following eigenvalue problem for the coefficients $q_{ki}$:

(14) $$Qq=\lambda^{2}Q,\;Q_{ij}=\frac{1}{N_{t}}\left(u_{i}\left(x\right),u_{j}\left(x\right)\right).$$

The dimension of this eigenvalue problem is equal to the number of snapshots, which is typically much lower than the dimension of the eigenvalue problem in Equation (6). The method of Sirovich uses the ergodicity hypothesis to approximate $R_{s}$; thus, we can expect the POD eigenfunctions to converge to the POD eigenfunctions of the continuous formulation. In fact, the snapshot method proves equivalence of information content in space and time correlation. This fact can be utilized for the analysis of both space and time structures, as applied in bi-orthogonal decomposition.

Please note that, in snapshot POD, the number degrees of freedom is equal to $N_{t}$.

### 3.4. Bi-Orthogonal Decomposition

Bi-orthogonal decomposition (BOD) represents an extension of POD; technically, it is a combination of the POD and snapshot POD methods. While POD analyzes data in the spatial domain only, BOD performs spatio-temporal decomposition. The effective number of degrees of freedom is now given by the complexity of the process in space and time. In practice, we consider N degrees of freedom,

(15) $$N=\min\left(N_{x},N_{t}\right),$$

as the complexity in both domains (space and time) should be equivalent.

Aubry [3] presented BOD as a deterministic analysis tool for complex spatio-temporal signals. Firstly, a complete two-dimensional decomposition was performed. These decompositions were based on two-point temporal and spatial velocity correlations. A set of orthogonal spatial (Topos) and temporal (Chronos) eigenmodes were computed to allow the expansion of the velocity field. The BOD method analyzes a deterministic space–time signal (e.g., velocity) u(x,t), which is decomposed in the following way:

(16) $$u\left(x,t\right)=\sum_{k}\lambda_{k}\;\bar{\varphi_{k}\left(x\right)}\;\psi_{k}\left(t\right).$$

The bar denotes a complex conjugate; $\varphi_{k}\left(x\right)$ are spatial eigenfunctions (Topoi), $\psi_{k}\left(t\right)$ are temporal eigenfunctions (Chronoses), and $\lambda_{k}^{2}$ are the common eigenvalues. Please note that both Topos and Chronos are dimensionless and orthonormal. The only quantity with physical dimension on the right-hand side of Equation (16) is the square root of the eigenvalue $\lambda_{k}^{}$, which poses the same physical dimension as the quantity on the left-hand side. Mathematical details of the BOD method can be found in Reference [3].

Orthogonal decomposition is optimal in the sense of a fast convergence of the expansion with a small number of terms. It should be noted that BOD introduces a time–space separation in the velocity field expansion. While classic orthogonal decomposition POD is based on full two-point space–time correlations and entails space- and time-dependent eigenmodes, BOD is closer to analytical and numerical studies, whereby the velocity field is naturally expanded over products of spatial functions and temporal functions.

The evaluation technique of the eigenfunctions uses the same mathematics as the POD does. In principle, the Fredholm integral equation can be written in the two forms for space and time correlation matrices. The eigenvalues are common for both problems, but the eigenfunctions differ, of course. For the time domain formulation, we get the following form (compare with Equation (6)):

(17) $$\int_{T}R_{t}\left(t,t^{\prime }\right)\;\psi \left(t^{\prime }\right)dt^{\prime }=\lambda^{2}\;\psi \left(t\right),$$

where correlation matrix $R_{t}$ stands for 

(18) $$R_{t}\left(t,t^{\prime }\right)=\int_{\Omega }u\left(x,t\right)\;u^{T}\left(x,t^{\prime }\right)\;dx.$$

The Topoi and Chronoses are related in the following way:

(19) $$\begin{array}{l} \varphi_{k}\left(x\right)=\frac{1}{\lambda_{k}}\sum_{i=1}^{N}u\left(x,t_{i}\right)\psi_{k}\left(t_{i}\right)\\ \psi_{k}\left(t\right)=\frac{1}{\lambda_{k}}\sum_{i=1}^{N}u\left(x_{i},t\right)\varphi_{k}\left(x_{i}\right) \end{array}$$

The decomposition allows us to study the energy and entropy of the fluid system, as well as its dynamical behavior. 

The spatial quantities characterize the distributions in space, while the temporal quantities characterize those in time. According to the developers of BOD, there is no real link between BOD and POD, since they are based on fundamentally different principles. In fact, BOD can be seen as a time–space symmetric version of the Karhunen–Loève expansion or, in other words, a combination of classical POD and snapshot POD. However, the main difference seems to be the assumptions on the analyzed signal, which only has to be square integrable for BOD, instead of square integrable, ergodic, and stationary for POD. BOD is a more general method, and the POD method should be considered for particular cases. Moreover, BOD is not derived from an optimization problem of the mean-square projection of the signal as in POD, although the method of calculation of BOD also leads to an eigenvalue problem of a correlation operator. The geometrical interpretation in state space, especially the principal axes of the ellipsoid, vanishes in the case of BOD.

Each mode consists of the energy contents (sum of energy of all local velocity components), the spatial mode (Topos), and the temporal mode (Chronos). The mode amplitude is equal to the square root of the energy. The modes are typically ordered according to decreasing energy content. The original series of snapshots can be fully reconstructed using the entire set of modes. Neglecting the high-order modes, the low-energy random noise can be removed, which can arise as a consequence of the process randomness, measurement/evaluation errors, or in connection with unresolved sub-grid structures in the flow.

Both Topoi and Chronoses form orthonormal bases. To study the embedded system dynamics, the Chronoses multiplied by the mode amplitude can be used to characterize the system evolution in time.

Typically, high-energy modes are periodical patterns. In this case, two modes are related to each periodical pattern very often shifted by a quarter of a period—see modes 5 and 6 in the example in Section 6. For analysis of the periodical aspects of such flows, reconstruction using two such modes is adequate.

To capture the time resolution of the process in a proper way and obtain representative Chronoses, the usual rules should be followed, including the Nyquist criterion. This means that the acquisition frequency should be higher than twice the maximal frequency of the process. If this condition is not kept, the frequency content of the time-dependent behavior is not reproduced properly because of the masking effect of aliasing. However, to study the details of the behavior in time, a much higher multiplier is necessary (5–10 instead of 2). Details can be found in Reference [4].

The modes are orthogonal and, thus, uncorrelated. The decorrelation of modes is a necessary condition for their independence, but is generally not sufficient. In practice, for signals random in time, this condition is sufficient; however, for periodically time-evolved signals, the condition is satisfied for two phases shifted by π/2 relative to each other. The consequence of the orthogonal feature, which is in fact non-physical, is a problem in the physical interpretation of the individual modes. The spectra of all Chronoses are generally dense for a broad-band process.

A few characteristics are defined to characterize the decomposition results. Energy and entropy distributions in time and Euclidian space can be evaluated. Energy distributions show distributions of the kinetic energy of the process. Entropy or, more precisely, information entropy is a measure of the uncertainty associated with the underlying random process and it is connected with the compression of information using the BOD method.

In the case of one of the space coordinates being chosen to be the state variable for BOD analysis, the modes represent dynamical behavior of the process not in time, but in space in a given direction. Two types of modes result, as in the classical BOD variant. One mode characterizes the system behavior for the chosen state variable (space coordinate) to be independent (Chronos in the classical variant), while the other involves all other space variables and time (Topos in the classical variant).

## 4. Energy and Entropy

The global energy of the system is represented by one half of the sum of variances of all quantities describing the system state. In our case, the quantities are all evaluated velocity components in all points in space. The space is always bounded, and not all velocity components are measured. In the PIV method, we typically have a measuring plane with regularly distributed measuring points forming a rectangular mesh. In all measuring points, the two in-plane velocity components are evaluated for classical PIV, and three components are evaluated in the case of stereo PIV. The eigenvalue $\lambda_{k}^{2}$ represent the *k*-th mode basic energy, while $\lambda_{k}^{}$ is the mode amplitude (see Equation (16)). The basic energy is defined as a sum of variances of all velocity components appearing in the given case.

Information entropy, or the Shannon–Kolmogorov entropy, represents in general the average rate at which information is produced by a stochastic source of data. The measure of information entropy associated with each possible data value is the negative logarithm of the probability mass function for the value: $S=-\sum_{i}p_{i}\ln p_{i}$ (e.g., see Reference [30]).

The global energy E can be evaluated as a sum of modes energies:

(20) $$E\left(u\right)=\frac{1}{2M}\int_{X}\int_{T}u_{i}\left(x,t\right)\bar{u_{i}\left(x,t\right)}\;dx\;dt=\frac{1}{2M}\sum_{k=1}^{N}\lambda_{k}^{2},$$

where the bar denotes a complex conjugate, and M is the number of points in Euclidian space.

The temporal energy $E_{t}\left(t\right)$ indicates the evolution of the system global energy in time:

(21) $$E_{t}\left(t\right)=\frac{1}{2M}\int_{X}u_{i}\left(x,t\right)\bar{u_{i}\left(x,t\right)}\;dx\;=\frac{1}{2M}\sum_{k=1}^{N}\lambda_{k}^{2}{\left|\psi_{k}\left(t\right)\right|}^{2}.$$

The spatial energy $E_{s}\left(x\right)$ indicates the distribution of the energy in space across the velocity components for the entire time interval *T* as follows:

(22) $$E_{s}\left(x\right)=\;\frac{1}{2M}\int_{T}u_{i}\left(x,t\right)\bar{u_{i}\left(x,t\right)}\;dt=\frac{1}{2M}\sum_{k=1}^{N}\lambda_{k}^{2}{\left|\varphi_{k}\left(x\right)\right|}^{2},$$

where *N* is the number of evaluated modes, and it is expected to be big. 

Please note that the following equation holds:

(23) $$E=\int_{T}E_{t}\;dt=\int_{X}E_{s}\;dx.$$

The spatial energy is equal to the TKE.

In a similar way, the information entropies can be evaluated. For this purpose, the probability of the individual mode appearance should be defined first.

As the Topoi and Chronoses are orthonormal, the probability of appearance of the given *k* mode could be put to its mean dimensionless amplitude *λ_k_* divided by the sum of amplitudes of all modes.

(24) $$p_{k}=\lambda_{k}^{}/\sum_{k=1}^{N}\lambda_{k}^{}.$$

Please note that the definition is different from those found in the literature, as entropy is based on amplitudes here, contrary to Reference [3], where a different definition is used based on variances.

The temporal evolution and spatial distributions of the velocity deviation probability are defined using the weighting of the modes as follows:

(25) $$\begin{array}{l} p_{tk}\left(t\right)=\lambda_{k}\left|\psi_{k}\left(t\right)\right|/\sum_{k=1}^{N}\lambda_{k}\left|\psi_{k}\left(t\right)\right|,\\ p_{sk}\left(x\right)=\lambda_{k}\left|\varphi_{k}\left(x\right)\right|/\sum_{k=1}^{N}\lambda_{k}\left|\varphi_{k}\left(x\right)\right|. \end{array}$$

Global entropy H, temporal entropy $H_{t}\left(t\right)$, and spatial entropy $H_{s}\left(x\right)$, with the help of their respective probabilities p, are defined as follows:

(26) $$H=-\frac{1}{\log N}\sum_{k=1}^{N}p_{k}\log p_{k},$$

(27) $$H_{t}\left(t\right)=-\frac{1}{\log N}\sum_{k=1}^{N}p_{tk}\left(t\right)\log p_{tk}\left(t\right),$$

(28) $$H_{s}\left(x\right)=-\frac{1}{\log N}\sum_{k=1}^{N}p_{sk}\left(x\right)\log p_{sk}\left(x\right).$$

We introduce the normalizing factor “log *N*” in order to perform comparisons between different signal entropies. The global entropy *H* is zero if and only if only just one eigenvalue is nonzero, i.e., all the signal energy is concentrated in the first mode. As usual, in the opposite case, if all the eigenvalues are equal, i.e., the energy is equally distributed among the modes, then *H* takes its maximum value, i.e., 1. At intermediate states, *H* keeps increasing as the amplitude spreads out uniformly on the eigenvalues. This function is useful in hydrodynamics for a quantitative description of the increasing degree of complexity as the Reynolds number (or another dimensionless parameter characteristic of the system, such as the Rayleigh number or an aspect ratio) gets higher and higher and the flow evolves toward a fully developed turbulent state and even beyond.

## 5. Energy and Entropy Discussion

The BOD energetic modes represent the dynamical content of the extended dynamical system under analysis. The modes are ordered in descending order, and the energy decay rate is a key feature of the BOD representation of system dynamics. The global entropy *H* can be used as an indicator of the modes’ decay rate. 

**Figure 1:**
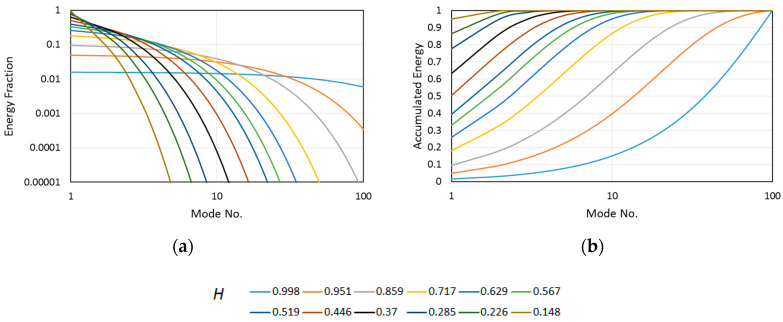
Simulated link between the global entropy and (**a**) the energy fraction, and (**b**) the accumulated energy.

To study the effect of the modes’ energy decay rate on the global entropy, the decay was modeled numerically for a low number of degrees of freedom equal to 100. The energy fraction across the modes is supposed to be close to exponential decay. In Figure 1, the energy fraction decays and accumulated energy curves are shown with corresponding global entropies, calculated using Equation (26).

The global entropy value ranges from 0.148 to 0.998. The value 0.998 means a nearly even distribution of the energy over the modes, resulting in nearly linear accumulated energy development. A lower global entropy value makes the convergence of the accumulated energy to 1 more effective. For *H* = 0.148, the first mode covers most of the system energy. Thus, the system dynamics could be modeled efficiently by a single mode. The evaluation of a given dynamical system in the context of ROM (e.g., see Reference [25]) can be performed with the help of global entropy. However, this information is irrelevant in the context of coherency and complexity of the given mode topology and/or its evolution in time. In general, as the mode increases in order, its energy decreases and its complexity increases.

In the future, the method of complexity of individual BOD mode evaluation is to be addressed.

## 6. Example

As an example, the case of analysis of turbulent wake behind an inclined flat plate is shown.

### 6.1. Experimental Set-Up

A flat plate inclined with an angle of attack of 7° was placed in a uniform low-turbulence stream. The blow-down facility produced a jet with uniform velocity distribution in the area 250 × 250 mm^2^, with a mean velocity of about 10 m/s, an intensity of turbulence of less than 0.2%, and mean velocity departures smaller than 0.5%. The plate of thickness 2 mm had rounded edges, a chord of 100 mm, and a span of 300 mm.

The used time-resolved PIV measuring system DANTEC consists of a laser with cylindrical optics and one charge-coupled device (CCD) camera with a lens of 60-mm focal length. The Laser New Wave Pegasus Nd:YLF with a double head produces light with a wavelength of 527 nm; the maximal repetition frequency of double-pulses is 10 kHz, and a shot energy is 10 mJ for 1 kHz (corresponding power of 10 W per head). The camera Phantom V611 with a resolution of 1280 × 800 pixels and a frequency of up to 3000 double-snaps per second was used; the internal memory was 8 GB. Tracing particles with a mean diameter of 1 µm were introduced to the flow upstream of the test section. To produce the particles, the SAFEX generator was used. Software Dynamic Studio ver. 3.4 was applied to data acquisition and post-processing. Interrogation areas were chosen to be 32 × 32 pixels, while the physical dimension of the measuring point was about 1.3 mm. Only the two in-plane velocity components were evaluated. Instantaneous velocity fields in the flow-domain were acquired with a frequency of 1 kHz; 4000 snapshots are available in a record.

**Figure 2:**
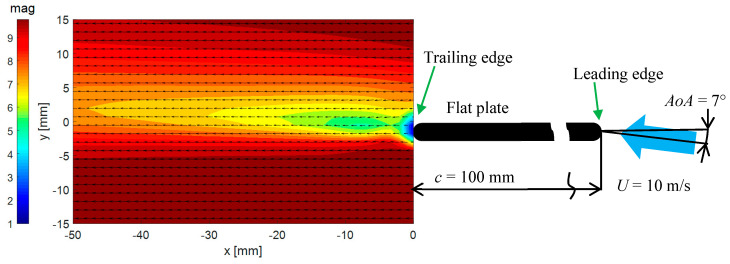
The mean velocity vectors and modulus distribution.

A schematic view of the experimental set-up is shown in Figure 2. The flow is from right to left.

The region in the wake was subjected to analysis, and the plate’s trailing edge was located in the coordinate system origin. The region of interest was behind the plate, which had a physical size of 50 × 30 mm^2^.

The experiment is described in more detail in Reference [31].

### 6.2. Results and Discussion

In Figure 2 the distributions of mean velocity vectors (arrows) and mean velocity magnitudes (color) are shown. The magnitude scale “mag” is given in m/s. 

**Figure 3:**
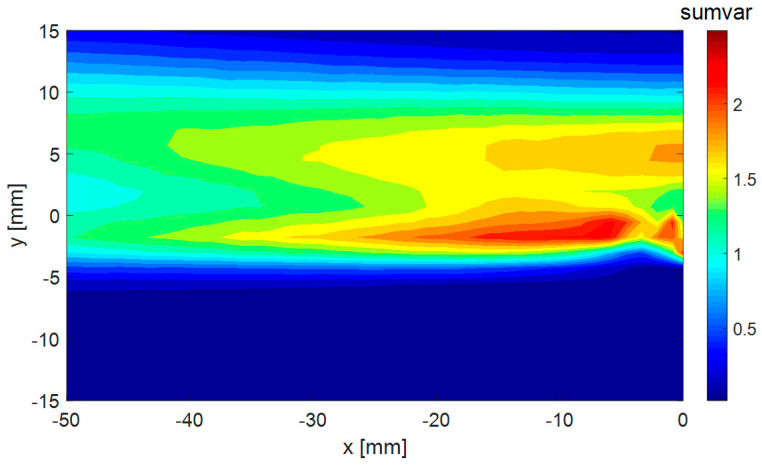
Distribution of sum of velocity component variances.

To estimate the velocity flow-field dynamics, variances of the velocity components at every point in space were evaluated. In Figure 3, a distribution of the evaluated sums of velocity component variances is shown, covering a part of the fluctuating kinetic energy at a given point. Please note that only two velocity components were evaluated (in the *x* and *y* directions). The “sumvar” scale (i.e., the sum of velocity component variances) is given in m^2^/s^2^.

The dynamic activity was maximal just behind the plate, and the secondary maximum was 5 mm above.

Next, the BOD method (see Section 3.4) was applied to the set of 4000 velocity distributions (“snapshots”) available. The energetic modes were evaluated together with the other relevant characteristics, across the 2340 modes, which was equal to the number of degrees of freedom (see Equation (15)), with a field of 26 × 45 vectors with two components each.

**Figure 4:**
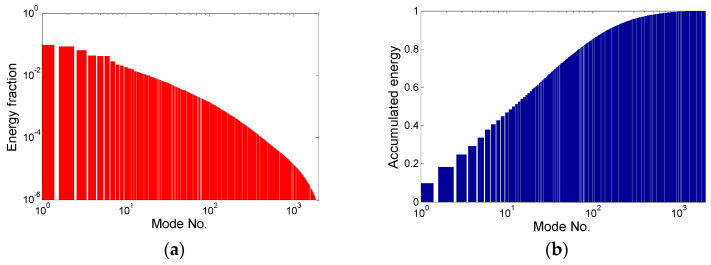
Energy distribution over the modes: (**a**) energy fraction; (**b**) accumulated energy.

Firstly, the energy distributions over the modes are shown. In Figure 4, the energy fraction and accumulated energy are depicted graphically. Both graphs represent dimensionless quantities. The energy fraction indicates the fraction of the total energy covered by the given mode. The first mode covers 9.7% of the total kinetic energy, the second mode covers 8.7%, the third mode covers 6.5%, etc. The modes are ordered in terms of descending energy content. The accumulated energy shows the energy content of the first *n* modes in sum. For example, the first 10 modes cover about 40% of the total energy. This information is important for the correct choice of mode truncation threshold for the sake of ROM.

Please note that the energy fraction is represented in logarithmic coordinates, while accumulated energy is represented semi-logarithmically.

Each energetic mode is represented by Topos, Chronos, and amplitude (or energy). A few examples of modes are shown and commented. Please note that the Topos reflects flow topology represented by the vector-field regardless of its amplitude, which could be virtually any value, positive or negative. The Chronos is represented by a signal over time *t*.

Topoi are depicted as black vectors with added vector-lines in red. Please note that arrows represent local velocity direction and dimensionless modulus, while vector-lines show overall vector-field structure, e.g., vortices.

**Figure 5:**
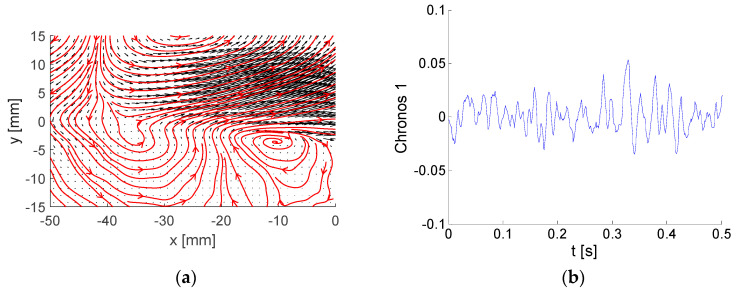
Example of bi-orthogonal decomposition (BOD) mode number (No.) 1: (**a**) Topos; (**b**) Chronos.

The first mode, containing 9.7% of the total kinetic energy, is documented in Figure 5.

The Topos (Figure 5a) is represented by intensive streamwise flow in the upper domain half, where the oscillations are negative and positive with low frequency defined by the corresponding Chronos (Figure 5b). The vortex located at [−10; −3] is relatively weak and, thus, not important from the energy point of view. Please note that only part of the Chronos is shown to demonstrate the signal structure, while the whole Chronos covers the time range of 0–4 s.

**Figure 6:**
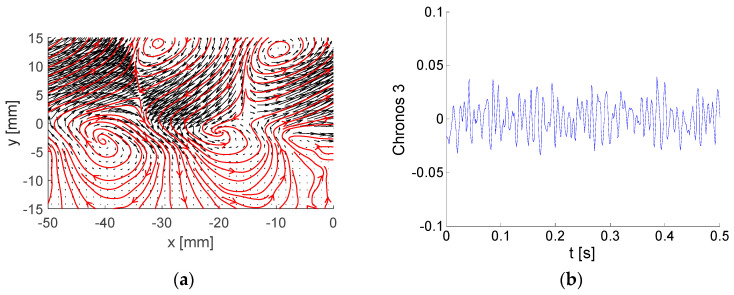
Example of BOD mode No. 3: (**a**) Topos; (**b**) Chronos.

The next example is mode number (No.) 3 covering 6.5 % of the total kinetic energy. The Topos and Chronos are depicted in Figure 6.

Two rows of big and strong counter-rotating vortices located in the upper half of the flow-field represent the corresponding Topos. The Chronos suggests higher-frequency contents of pulsations in comparison with mode 1.

**Figure 7:**
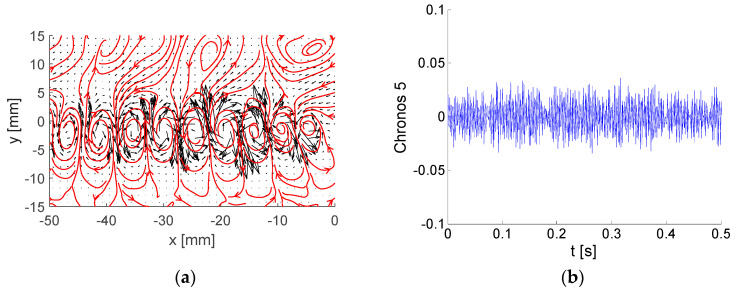
Example of BOD mode No. 5: (**a**) Topos; (**b**) Chronos.

**Figure 8:**
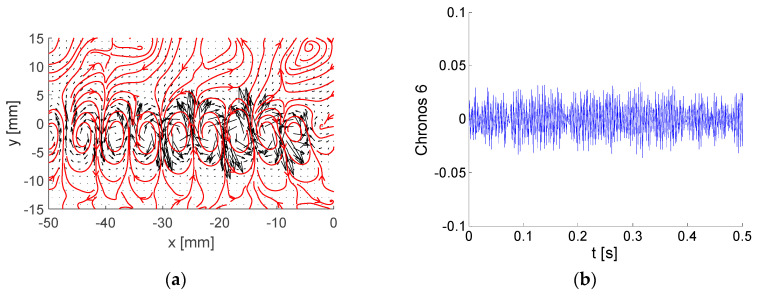
Example of BOD mode No. 6: (**a**) Topos; (**b**) Chronos.

Modes No. 5 and 6 are very similar to each other (see Figure 7 and Figure 8). The energy fractions are 4.27% and 4.23% of the total kinetic energy, respectively; altogether, these two modes contain 8.5%. The Topoi represent a common flow pattern: vortex-street behind the plate, which is a periodical process both in space and time. The difference between the modes is in phase, with modes 5 and 6 shifted both in space and time by ¼ of a period. This means that the positions of vortices in Topoi are shifted in space, while Chronoses are shifted in time. The frequency is around 390 Hz, obviously much higher than in the preceding modes.

**Figure 9:**
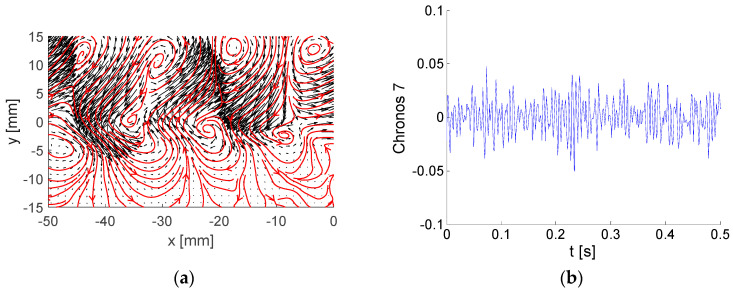
Example of BOD mode No. 7: (**a**) Topos; (**b**) Chronos.

The last example is mode No. 7, containing 2.8% of the global kinetic energy in Figure 9. Again, as for mode No. 3, we can identify another configuration of the two rows of the counter-rotating vortices. However, the vortex topology is different, as they are smaller than in the case of mode 3 (compare with Figure 6a).

Generally, as the mode order increases, the energy decreases, smaller structures are involved in the corresponding Topos, and higher frequencies are involved in the corresponding Chronos. However, if periodical structures are represented, the energy is higher and the mode order is lower than expected.

**Figure 10:**
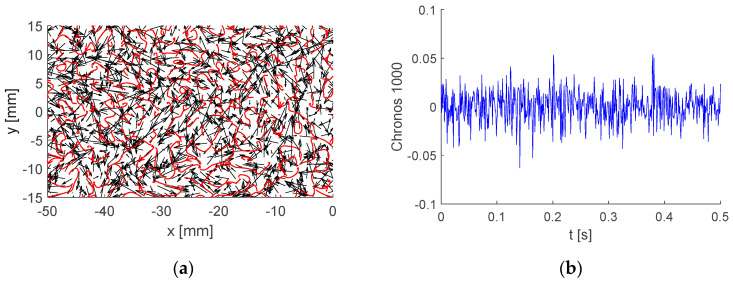
Example of BOD mode No. 1000: (**a**) Topos; (**b**) Chronos.

To demonstrate this tendency, mode No. 1000 is shown in Figure 10.

Both Topos and Chronos of BOD mode 1000 show predominantly random features. Its energy is extremely low, with only 0.0013% of the global kinetic energy.

**Figure 11:**
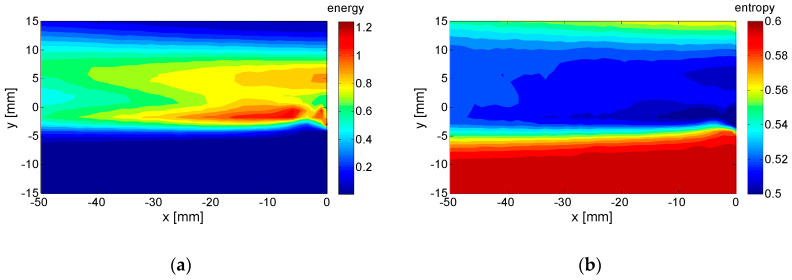
Distributions in space: (**a**) energy; (**b**) entropy.

**Figure 12:**
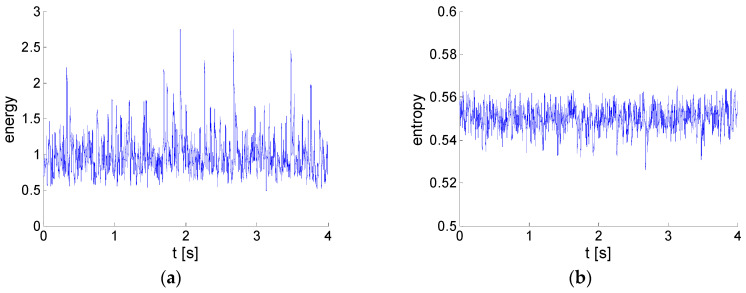
Distributions in time: (**a**) energy; (**b**) entropy.

Finally, the global energy and entropy distributions in space and time were evaluated (see Section 4) for the given case. In Figure 11, there distributions in space are shown, while Figure 12 presents the distributions in time.

The global energy and entropy were evaluated using Equations (20) and (26): E = 0.725 m^2^/s^2^; *H* = 0.550. The normalization of temporal and spatial energy and entropy makes the global values the mean both in space and time.

The distributions of energy and entropy in space are close to being opposite to each other. In the wake region, there is a maximum of energy, while entropy is minimal. This means that the velocity variance in the wake could be predominantly linked to coherent structures. On the other hand, the flow in the lower half of the flow-field consists of low-energy highly random structures. The randomness could come from the fact that the low-level signal is subjected to an important relative random error of measurement. The random error of the PIV measurement could be estimated as about 2% of maximal velocity in the whole field. In the lower part of the domain, for y < −5 mm, the entropy is high, because the coherent fluctuations are negligible and, thus, the random part prevails. The distributions in time in Figure 11 indicate highly randomly distributed peaks in the energy signal, while the entropy time evolution is relatively less disturbed.

**Figure 13:**
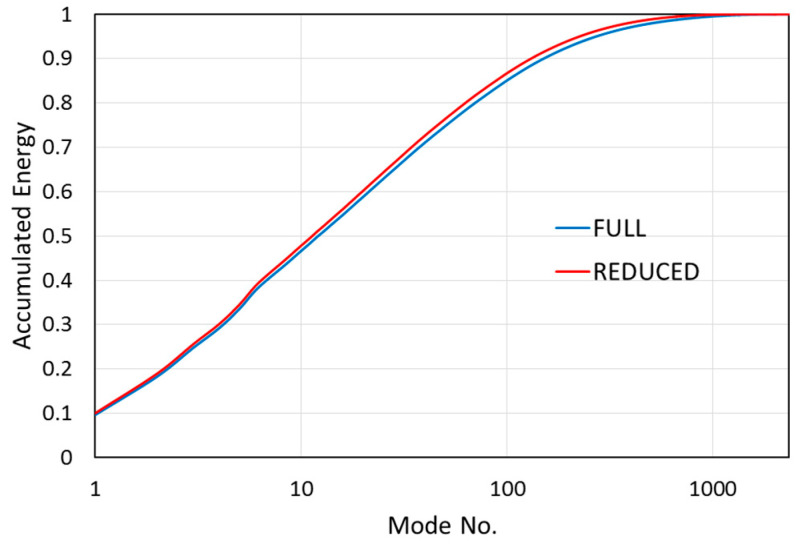
Comparison of accumulated energy for the full and reduced areas of interest.

The information from the energy and entropy space distribution can be used for the optimization of dynamics modeling using the ROM method. To get lower global entropy and, thus, better coherence and better compression of the information, the space domain should be limited to regions with low spatial entropy. In our example, a reduction of the *y* range to (−5; 13) mm seems to be a good choice. Indeed, the global entropy is, in this case, close to 0.5, and the accumulated energy approaches full energy (1) faster—see Figure 13.

**Figure 14:**
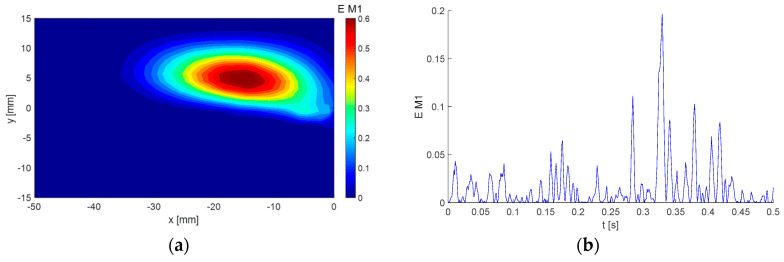
Energy of the first BOD mode distributions (**a**) in space, and (**b**) in time.

Next, we studied the selected individual modes or their combinations. Firstly, we considered the global energy. Please compare Figure 10a, showing the distribution of the energy in space, with Figure 3, representing the distribution of the sum of velocity component variances. The distributions are identical, whereby the values of energy are half the values of the sum of velocity component variances. This is consistent with the energy definition in Equation (22). An evaluation of the energy distribution in space using the BOD technique can be considered as a very expensive way of evaluating the energy distribution in space. However, the procedure can be used for evaluation of the energy distribution connected only with a specific BOD mode or modes. To demonstrate this feature, the energy distributions linked to the first BOD mode were evaluated and are presented in Figure 14.

The energy of the first mode is located in the upper part of the flow-field. The time evolution indicates very intensive bursts randomly distributed in time followed by calm periods (energy approaching 0) in between.

**Figure 15:**
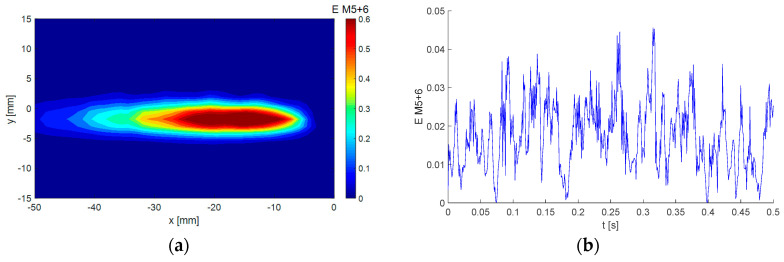
Sum of energies of the fifth and sixth BOD modes distributions (**a**) in space, and (**b**) in time.

A very different energy distribution in time is shown in Figure 15b, where the two modes 5 and 6 representing the vortex shedding process are involved (see Figure 6 and Figure 7). The energy level is lower than for BOD 1; however, it is more uniform, and no bursts are present. The resulting global energy is nearly the same. This behavior corresponds to a quasi-periodical process. The energy is concentrated in the wake close to *y* = 0.

Compare the time evolution of the energy of modes 5 and 6 in Figure 15b with the Topoi of corresponding modes 5 and 6 in Figure 7b and Figure 8b. The sum of energies in Figure 15b represents the square of the amplitude corresponding to the vortex shedding process involved in BOD modes 5 and 6.

Generally, the global energy can be decomposed on the basis of BOD modes.

## 7. Conclusions

Definitions of energy and entropy were given in this paper in the context of turbulent velocity field decomposition. The POD and BOD methods were implemented accordingly.

The mode energy quantifies the significance of the individual mode. Topos and Chronos characterize distributions of the given mode energy in space and time, respectively.

The global entropy is the factor determining the spatio-temporal data coherency; it measures coherency of the entire system. Temporal entropy shows the distribution of entropy over time, while spatial entropy shows the distribution of entropy in space. This information, together with energy distributions, is helpful in the physical interpretation of energetic modes.

Examples of distributions of energy and entropy both in space and time were also shown. Generally, the entropy is higher for the low-energy regions, where the random signal prevails. High-energy structures are coherent as a rule.

Global entropy seems to be a good measure of the reduced-order modeling technique’s efficiency. An evaluation method of the coherency of individual modes is to be developed in the future.
